# Second malignant neoplasms within 5 years from first primary diagnosis in pediatric oncology patients in Canada: a population-based retrospective cohort study

**DOI:** 10.3389/fonc.2024.1376652

**Published:** 2024-03-28

**Authors:** Christina Ricci, Divya Subburaj, Kate Lim, Neetu Shukla, Jaskiran Kaur, Lin Xie, Meghan Laverty, Dianne Zakaria, Jason Pole, Marie-Claude Pelland-Marcotte, Randy Barber, Sara J. Israels, Thai-Hoa Tran, Sapna Oberoi, Samuele Renzi, Tamara MacDonald, Lillian Sung, Ketan Kulkarni

**Affiliations:** ^1^ Lifespan Chronic Disease and Conditions Division, Public Health Agency of Canada, Ottawa, ON, Canada; ^2^ Department of Pediatrics, Division of Hematology-Oncology, Izzak Walton Killam (IWK) Health Centre, Halifax, NS, Canada; ^3^ Faculty of Medicine, Dalhousie University, Halifax, NS, Canada; ^4^ Surveillance Systems and Data Management Division, Public Health Agency of Canada, Ottawa, ON, Canada; ^5^ Dalla Lana School of Public Health, University of Toronto, Toronto, ON, Canada; ^6^ Centre for Health Sciences Research, University of Queensland, Brisbane, QLD, Australia; ^7^ Division of Pediatric Hematology-Oncology, CHU de Québec-Centre Mère-Enfant Soleil, Quebec City, QC, Canada; ^8^ Research Centre of the CHU de Québec, Axe Reproduction, Santé de la Mère et de l’Enfant, Quebec City, QC, Canada; ^9^ C17 Research Network, C17 Council, Edmonton, AB, Canada; ^10^ Department of Pediatrics and Child Health, University of Manitoba, Winnipeg, MB, Canada; ^11^ Department of Pediatrics, Division of Pediatric Hematology-Oncology, Charles-Bruneau Cancer Center, Centre Hospitalier Universitaire (CHU) Sainte-Justine, Montréal, QC, Canada; ^12^ Immune Diseases and Cancers Axis, CHU Sainte-Justine Research Center, Montréal, QC, Canada; ^13^ Department of Pediatric Hematology-Oncology, CancerCare Manitoba, Winnipeg, MB, Canada; ^14^ Division of Hematology Oncology, The Hospital for Sick Children, Toronto, ON, Canada; ^15^ Department of Anesthesia and Intensive Care, IRCCS San Raffaele Scientific Institute, Milan, Italy; ^16^ Department of Pharmacy, Izzak Walton Killam (IWK) Health, Halifax, NS, Canada; ^17^ Faculty of Health Professions, Dalhousie University, Halifax, NS, Canada; ^18^ Child Health Evaluative Sciences, Research Institute, The Hospital for Sick Children, Toronto, ON, Canada

**Keywords:** oncology, pediatrics, risk factors, early second malignant neoplasm (early SMN), Canada, surveillance

## Abstract

**Introduction:**

From the advancement of treatment of pediatric cancer diagnosis, the five-year survival rate has increased significantly. However, the adverse consequence of improved survival rate is the second malignant neoplasm. Although previous studies provided information on the incidence and risk of SMN in long term survivors of childhood cancer, there is still scarce information known for short term (< 5 years) prognosis. This study aims to assess the incidence, characteristics, management, and outcome of children who develop SMN malignancies within 5 years of diagnosis of their initial cancer.

**Method:**

This is a retrospective cohort study of early Second Malignant Neoplasms (SMN) in pediatric oncology patients. The Cancer in Young People – Canada (CYP-C) national pediatric cancer registry was used and reviewed pediatric patients diagnosed with their first cancer from 2000-2015.

**Results:**

A total of 20,272 pediatric patients with a diagnosis of a first malignancy were analyzed. Of them, 0.7% were diagnosed with a SMN within the first 5 years following their first cancer diagnosis. Development of a SMN impacted survival, shown by an inferior survival rate in the SMN cohort (79.1%) after three years compared to that of the non-SMN cohort (89.7%). Several possible risk factors have been identified in the study including the use of epipodophyllotoxins, exposure to radiation, and hematopoietic stem cell 169 transplant.

**Discussion:**

This is the first national study assessing the incidence, 170 characteristics, risk factors and outcome of early SMN in Canadian children 171 from age 0-15 from 2000-2015.

## 1 Introduction

The five-year survival of children following a cancer diagnosis currently exceeds 84% (1). However, survivors of childhood cancer still experience a diverse spectrum of short-term and long-term complications (1). Second malignant neoplasms (SMN) are one such adverse outcome and pediatric cancer survivors carry a higher risk of developing SMN compared to the general population (2, 3). Fortunately, there has been a significant improvement in the long-term mortality outcomes for five-year cancer survivors, with a decrease in 15-year overall mortality rates from 10.7% to 5.8% in the current era (4). As the five-year cancer survival rate increased, it became evident that the long-term survivors of childhood cancer were at increased risk for severe treatment-related late effects (4). Unfortunately, this late effect may be associated with reduced survival rate of those who develop a second malignant neoplasms as reported by the inferior five-year relative survival rate in this population compared to those who did not develop SMN (3). It is noted that Armstrong et al. described reductions in late SMN specific deaths over their study period (4). Furthermore, the inferior survival rate of those who developed SMN was associated with the reduction in therapeutic radiation dose (5).

These current epidemiological data have helped with the development of guideline recommendations for surveillance and strategies to reduce therapeutic exposure, both of which have played a vital role in improving the short-term and long-term outcomes among five-year survivors of their first cancer (6–11). A study that followed patients for up to 26.4 years demonstrated that about 40% of SMNs are diagnosed in the first 5 years after a first primary malignancy (12). However, acknowledging that the SMN incidence does not plateau over time, studies describing this cohort of early-onset SMN are limited (12). Currently, extensive data and literature exist for specific follow-up after initial childhood cancer. For example, breast cancer screening among survivors at risk begin at 8 years after radiation or at age 25, whichever occurs last (13) and colorectal cancer screening begins 5 years after radiation or at age 30, whichever occurs last (14). Additionally, other potential subsequent neoplasms, such as t-AML after exposure to epipodophyllotoxins, alkylating agents and anthracyclines are meant to be screened annually for 10 years post-exposure (14). These guidelines are based on extensive literature review and updated every few years. However, additional strategies for surveillance of early onset SMN are required to identify those with early presentation of SMNs in the first 5 years following diagnosis of their first primary malignancy (3, 15) because poorer outcomes are seen in patients with early onset SMNs (2, 3, 15). Nearly half of the non-relapse causes of mortality among the five-year first primary cancer survivors can be the result of SMNs (16, 17).

The primary aim of this national population-based surveillance study was to assess the cumulative incidence, clinical characteristics, and outcomes of pediatric patients with a diagnosis of SMN within 5 years of the first primary cancer diagnosis. The secondary aim was to analyze potential risk factors for SMN within the first five years using a proportional sub-distribution hazards regression model.

## 2 Methodology

### 2.1 Data sources

The primary data source used for this retrospective cohort study is the Cancer in Young People in Canada (CYP-C), a national pediatric cancer surveillance database. Since January 1, 2001, the CYP-C has captured demographic and clinical information on cancers diagnosed and treated at one of the 17 pediatric oncology centers across Canada prior to the age of 15 years, and 18 years since January 1, 2015. Patient data were abstracted by Clinical Research Associates at each pediatric oncology center and submitted to the CYP-C database.

All eligible patients aged 0-18 years who were diagnosed with a primary malignancy from 2001-2019 in Canada were included in the study to have the largest sample size possible. The last date of follow-up was February 28, 2021, and data for this study were extracted on March 3, 2021. The CYP-C privacy rules entail that cases under five are suppressed and not published to protect participant identity.

### 2.2 Cohort and cancer diagnosis

The types of cancer were defined according to the International Classification of Childhood Cancer, Third Edition (ICCC-3) criteria, which is based on the World Health Organization’s ICD-O-3 cancer site and morphology coding definitions from 2008 (18). All neoplasms with any ICD-O-3 Behavior code were included in the analysis including *in-situ* neoplasms. Patient sociodemographic data captured in the database included sex, age at diagnosis, ethnicity, and national neighborhood income quintile. Neighborhood income quintiles were assigned using Statistics Canada’s Postal Conversion File Plus (PCCF+) and rural/urban status were assigned using the child’s residential postal codes captured at the time of diagnosis (19, 20). We denoted if someone lived in a rural setting based on the presence of “0” as the second character on the child’s Forward Sortation Area (FSA). According to Statistics Canada, the presence of a zero in the second position of the FSA code identifies the rural postal codes (20). To define the income quintiles, census data were used to rank dissemination area-level average household income into quintiles ranging from least (Quintile 1) to most affluent (Quintile 5), adjusting for household size, cost of living, and regional differences. Further details can be found in previous Statistics Canada documentation (16). Genetic predisposition was defined as any predisposing condition (e.g., ataxia-telangiectasia, DICER1 syndrome, etc.), comorbidity which modifies therapy, genetic condition, and various other conditions such as neurofibromatosis and Li-Fraumeni Syndrome. This genetic predisposition information was collected for all provinces with the exception of Ontario.

A SMN was defined as a cancer diagnosed after 60 days but within the first 5 years of the initial cancer diagnosis, that was histologically or morphologically distinct from the first primary cancer. Subsequent malignancies were determined to be histologically or morphological distinct from the primary cancer using morphology and topography codes in the CYP-C dataset. Since comprehensive examination at the first diagnosis can artificially inflate the risk of SMN in the first 60 days following a diagnosis, cancer diagnoses within 60 days of the first diagnosis were considered part of the initial diagnosis. This 60-day window has been used in other studies to decrease the risk of differential surveillance (16, 21, 22) into one of the seven main diagnostic types of childhood cancer (**Table 1**) to mitigate small numbers of events. Only the first SMN was used to indicate a SMN as there were very few cases with multiple SMNs.

**Table 1:** Characteristics of patients first diagnosed with cancer at age 0 to 18 years, between 2001 and 2019 in Canada, by SMN status during five-year follow up.

Characteristic	Overall Cohort		SMN				Proportion of SMN vs. Total sample size
			No		Yes		
	Rounded N^b^	%	Rounded N^b^	%	Rounded N^b^	%	%
Total	20,272	100	20,138	100	134	100	0.66
Age at initial diagnosis							
0-4	8,660	42.73	8,620	42.79	45	33.58	0.52
5-9	4,765	23.51	4,735	23.51	30	23.13	0.65
10-14	4,890	24.14	4,850	24.06	50	35.07	0.96
15-18	1,950	9.63	1,940	9.64	10	8.21	0.56
Sex							
Male	11,065	54.58	10,990	54.58	75	55.22	0.67
Female	9,205	45.42	9,145	45.42	60	44.78	0.65
Population group							
Asian	1,935	9.56	1,920	9.54	15	11.19	0.77
White	11,010	54.32	10,930	54.27	85	62.69	0.76
Other Non-White	3,730	18.39	3710	18.43	20	13.43	0.48
Not available	3,590	17.73	3,575	17.76	20	12.69	0.47
Province or Territory of residence							
Atlantic Canada (Newfoundland, Prince Edward Island, Nova Scotia, New Brunswick)	1,170	5.77	1,160	5.78	10	5.22	0.60
Quebec	4,060	20.02	4,035	20.04	25	17.16	0.57
Ontario	9,525	46.98	9,450	46.94	70	52.99	0.75
Western Canada (Manitoba, Saskatchewan)	1,290	6.37	1,280	6.37	10	6.72	0.70
Alberta	1,990	9.81	1,970	9.77	20	15.67	1.06
British Columbia	2,045	10.1	<2045^a^	<10.5^a^	<5	ƚ	
Territories (Nunavut, Northwest Territories, Yukon)	50	0.25	<55^a^	<0.3^a^	<5	ƚ	
Missing	140	0.71	<145^a^	<0.75^a^	<5	ƚ	
Geographic location							
Urban	16,585	81.81	16,470	82.54	115	87.31	0.71
Rural	3,500	17.27	<3490^a^	<17.5^a^	<20 a	<12.8 ^a^	
Missing	185	0.92	<190^a^	<0.95^a^	<5	ƚ	
Neighborhood income quintile							
Lowest income quintile	3,620	17.87	3,600	17.88	20	16.42	0.61
Second income quintile	3,575	17.65	3,545	17.61	30	22.39	0.84
Third income quintile	3,830	18.9	3,800	18.87	30	23.13	0.81
Fourth income quintile	4,140	20.42	<4120^a^	<20.5^a^	<30^a^	<21 a	
Highest income quintile	4,020	19.83	4,000	19.86	20	14.93	0.50
Missing	1,080	5.33	<1080^a^	<5.5^a^	<5	ƚ	
Calendar period of diagnosis							
2001-2004	3,810	18.79	3,775	18.75	35	25.37	0.89
2005-2009	5,040	24.88	5,000	24.83	40	31.34	0.83
2010-2014	5,510	27.2	5,475	27.18	40	29.1	0.71
2015-2019	5,910	29.14	5,890	29.24	20	14.18	0.32
Treatment era*							
2001-2010	8,175	40.33	8,120	40.32	55	41.79	0.69
2010-2019	10,675	52.65	10,625	52.76	50	36.57	0.46
No treatment reported	600	2.96	580	2.88	20	15.67	3.50
Missing	820	4.06	815	4.05	10	5.97	0.97
Initial Cancer type							
Leukemia	6,100	30.1	6,060	30.1	40	29.85	0.66
Lymphoma	2,770	13.68	2,760	13.71	15	9.7	0.47
Central Nervous System	4,610	22.74	4,590	22.77	25	17.16	0.50
Bone tumors	870	4.3	860	4.26	15	10.45	1.61
Soft tissue sarcoma	1,210	5.95	1,195	5.94	10	7.46	0.83
Other/not specified cancer	4,560	22.51	4,535	22.52	30	20.9	0.61
Not classified by ICCC or *in-situ*	150	0.73	140	0.7	5	4.48	4.08
Radiation therapy							
Yes	5,190	25.62	5,140	25.52	55	40.3	1.04
No	15,080	74.38	14,995	74.48	80	59.7	0.53
Chemotherapy							
Yes	14,70	72.85	14,665	72.82	105	77.61	0.70
No	5,500	27.15	5,470	27.18	30	22.39	0.55
Chemotherapy drug agents**							
Alkylating agents and other	4940	24.38	4905	24.36	35	27.61	0.75
Alkylating agents, Epipodophyllotoxins and other	4390	21.67	4350	21.6	40	32.09	0.98
Epipodophyllotoxins and Other	1080	5.34	1070	5.32	10	8.21	1.02
Missing chemotherapy agent	<5	ƚ	<5	ƚ	<5	ƚ	
No chemo agent given	5830	28.77	5800	28.81	30	23.13	0.53
Other	<4030	19.85	<4015	19.92	<20	8.96	0.30
Radiation dose (cumulative)							
Did not get radiation	15080	74.38	14995	74.48	80	59.7	0.53
>0 to <3,000	1290	6.36	1275	6.34	10	8.96	0.93
>=3,000 to <6,000	1040	5.14	1030	5.11	10	8.21	1.06
>=6,000	130	0.64	<130^a^	<0.65^a^	<5	ƚ	
Missing Dose	2730	13.48	<2710	13.43	<35	20.9	1.02
Alkylating dose (cumulative) (mg/m^2^)							
>0 to <4,000	5330	26.28	5290	26.27	35	27.61	0.69
>=4,000 to <8,000	1560	7.69	1550	7.7	10	6.72	0.58
>=8,000	2450	12.08	2415	11.99	35	25.37	1.39
Did not receive alkylating dose	10940	53.96	10880	54.05	55	40.3	0.49
Genetic predisposition							
Yes	3,470	17.12	3,435	17.06	35	26.87	1.04
No	16,800	82.88	16,700	82.94	100	73.13	0.58
HSCT***							
Yes	1,945	9.6	1,920	9.54	25	17.91	1.23
No	18,325	90.4	18,220	90.46	110	82.09	0.60
Surgery							
Yes	9,570	47.21	9,500	47.18	70	51.49	0.72
No	10,700	52.79	10,635	52.82	65	48.51	0.61
Registered in a clinical trial							
Yes	4,950	24.43	4,915	24.42	35	26.87	0.71
No	15,320	75.57	15,220	75.58	100	73.13	0.65
Outcomes****							
Relapse	2,485	12.26	2,455	12.2	30	21.64	1.17
Death	3,120	15.39	3,060	15.2	60	44.03	1.89
SMN	134	0.66	-	-	–	–	

*Treatment era includes the number of children that had at least one of the following levels of treatment prior to or after 2010: surgery alone, radiation alone, surgery and radiation, having individualized treatment, standard of care, under observation alone, under observation alone and not on a clinical trial, following a protocol, registered on a clinical trial protocol, registered on a clinical trial that is research ethics board approved, following a clinical trial that is research ethics board approved but not enrolled, standardized regimen and other not listed.

***HSCT includes both autologous and allogenic hematopoietic cell transplantation due to the classification not always being defined in the original data set.

**The other category includes: Anthracyclines, Antimetabolite, Purine/pyrimidine analogue, Topoisomerase-2 inhibitors, Platinum.

****Values will not equate to the sum of the total cohort as children could have experienced a combination of death, relapse and SMN outcomes.

ƚSuppressed because proportion is based on less than 5 people in the numerator.

a Estimate was suppressed to prevent disclosure through differencing.

b To ensure confidentiality, case counts are randomly rounded either up or down to a multiple of 5. This aligns with Statistics Canada’s guidance on rounding (23).

SMN, Second malignant neoplasm; N, Sample Size; ICCC, International Classification of Childhood Cancer; HSCT, hematopoietic stem cell transplantation.

A very-early onset SMN was defined as a SMN that occurred more than 60 days but within 2 years of the initial cancer diagnosis, and an early SMN defined as one that occurred between 2-5 years from the first primary cancer diagnosis.

### 2.3 Treatment information

All treatment related information including surgery, radiation, hematopoietic stem cell transplant (HSCT), and chemotherapy was obtained from the CYP-C. The treatment era presented in **Table 1** distinguishes patients who had received any treatment prior to 2010 from those who received treatment in 2010 or later. This categorization accounts for treatment advancements that occurred in childhood cancer after 2010, which have helped reduce the incidence of SMNs (24, 25). All treatment variables were modeled as dichotomous except for type of chemotherapy, cumulative dose of alkylating agent, and cumulative dose of radiation.

There were six main groupings for the chemotherapeutic agents included in the study, as seen in **Table 1**. The alkylating agents’ cumulative dose was captured for those who had an alkylating agent as part of their treatment. Cumulative doses (mg/m^2^) of alkylating agents were categorized as: 1) did not receive alkylating agents; 2) greater than zero to less than 4,000; 3) greater than or equal to 4,000 to less than 8,000; 4) and greater than or equal to 8,000 (26).

The cumulative radiation doses in centigray (cGy) were categorized as: 1) did not receive radiation therapy; 2) greater than 0 to less than 3,000; 3) greater than or equal to 3,000 but less than 6,000; 4) greater than or equal to 6,000 cGy; and 5) missing cumulative radiation dose.

### 2.4 Statistical analyses

All statistical analyses were performed using SAS Enterprise Guide 7.1 (27). Proportions for first primary cancers and SMNs were based on cancers registered in the CYP-C, similarly, grouped by demographic and treatment factors.

To assess the overall risk of death among our SMN cohort, regardless of competing risks, a cox proportional hazards curve was used to calculate the probability of survival over time among the CYP-C patients. We used the cumulative incidence function to estimate the cumulative probability of being diagnosed with a SMN with (28) 95% confidence intervals estimated according to Hosmer, Lemeshow, and May method (29). A proportional sub-distribution hazards regression model was developed to examine the factors associated with the development of a SMN in the presence of the competing event of death (30, 31). For the competing risk model, participants in the CYP-C contributed person-time from the date of initial diagnosis until the earliest of either when they were diagnosed with a second cancer, died, reached the end of their five-year follow-up or February 28, 2021 for those who did not have a full 5 years of follow-up yet. The sub-distribution hazard function treats the event of interest mutually exclusive from the competing risks and the hazard of the event of interest is adjusted for the cumulative incidence of the competing events. This was done using the %PSHREG SAS macro (31). We developed three models to examine cancer occurrences at different time periods from the first cancer diagnosis. The first model examined all SMNs within the first 5 years, the second model examined very-early SMNs diagnosis, and the last model included the early SMNs. To determine which variables to account for, we decided on several variables *a priori*, and then used a stepwise model building approach at the 0.05 level to decide on the variables that would be used as confounders in the model. We initially considered sex, age at diagnosis, calendar period of diagnosis, treatment era, population group, first primary cancer type, urban/rural status, neighborhood income quintile, province or territory of residence and various treatment options and dosages. If the removal of a variable considerably changed the estimates for the remaining variables by more than 10%, then that variable was retained. Despite this, we kept chemotherapy agent and HSCT given that previous studies have shown these variables as risk factors for SMN development (12, 32–34). Values were reported as hazard ratios (HR) with 95% confidence intervals.

## 3 Results

A total of 20,272 pediatric patients with a diagnosis of a first malignancy were analyzed during the study period from 2001 to 2019 (**Table 1**). From these, 134 (0.7%) were diagnosed with a SMN within the first 5 years following their first cancer diagnosis.

### 3.1 Characteristics of the first primary cancer cohort

Relative to the cohort without a SMN, those treated in earlier study time periods were more likely to develop SMNs. The period from 2015-2019 had the lowest number of the SMN cases diagnosed; however, this may be due to early censoring of the study data as we do not have the 5-year follow-up for those diagnosed in 2019. The majority of the patients with first malignancy and SMN came from an urban setting. There were no major differences among the SMN and non-SMN groups with respect to income backgrounds. We found the proportion of children and youth with cancer who developed a SMN in Alberta was higher (1.1%) compared to the other provinces; however, the reason remains unclear and could be an artifact of responsive reporting of SMNs in Alberta (**Table 1**).

Among children with SMN, the most common therapies used to treat the first malignancy were again chemotherapy (77.6%), surgery (51.5%) and radiation (40.3%). Among those who had chemotherapy in the SMN cohort, 59.7% had been exposed to alkylating agents, and 40.3% to epipodophyllotoxins. An underlying genetic cancer predisposition was identified in 17.1% of pediatric patients with a first primary cancer diagnosis. Also, 26.9% of patients who developed a SMN were diagnosed with a cancer predisposition syndrome (**Table 1**). Since there was no genetic cancer predisposition information for Ontario, this is likely an underestimation of data.

### 3.2 Characteristics of the SMN cohort

**Figure 1:**
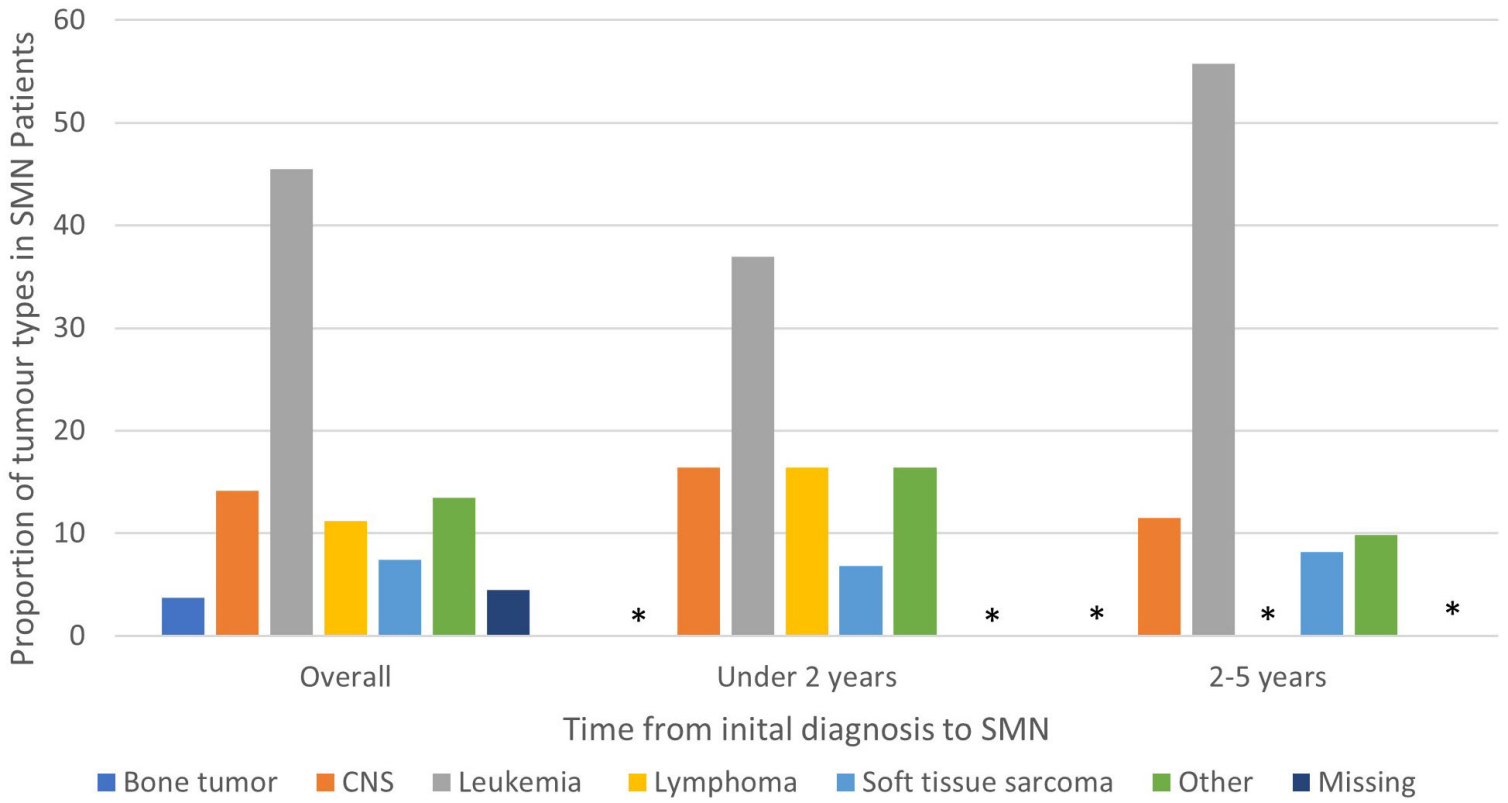
Types of SMN in overall cohort (5 years from diagnosis of first primary malignancy), 0-2 years and 2-5 years from diagnosis of first primary malignancy. Star means number not shown since less than 5.

Among the 134 patients diagnosed with SMN, 73 (54.5%) children developed a very-early onset SMN within 2 years of their first cancer diagnosis and 61 (46.5%) had an early onset SMN diagnosed between 2-5 years after their first cancer diagnosis. Leukemia (45.5%) was the most common SMN diagnosis within the first 5 years of their first cancer diagnosis (**Table 2**) CNS malignancy was the second most common SMN (14.2%) (**Figure 1**). 10.5% of those who developed a SMN had a prior diagnosis of a bone tumor, mostly Ewing sarcoma. This means that 1.6% of those whose first cancer was a bone tumor developed a SMN (**Table 1**). Leukemia was the most common second malignancy in both the very-early onset and early onset SMN cohorts (**Figure 1**) and acute myeloid leukemia was the most common type of leukemia constituting 85.0% of leukemia diagnoses in the SMN cohort [data not shown]. Of the 40 cases of leukemia who developed an SMN, 21 developed a secondary leukemia, 11 as very-early SMNs and 10 as early SMNs. Chemotherapy was the most common therapeutic modality used in the management of SMN (Appendix 1). Within the very-early SMN cohort, chemotherapy was used in 69.9% of patients and within the early SMN cohort it was used in 86.9% [data not shown].

**Table 2:** Types of SMN in overall cohort (5 years from diagnosis of first primary malignancy), 0-2 years and 2-5 years from diagnosis of first primary malignancy.

SMN diagnosis	Overall Rounded N= 134 (%)	Within 2 years from initial diagnosis Rounded N= 73 (%)	2-5 years from initial diagnosis Rounded N= 61 (%)
Bone Tumor	5 (3.7%)	*	*
CNS	20 (14.2%)	10 (16.4%)	10 (11.5%)
Leukemia	60 (45.5%)	25 (37.0%)	35 (55.7%)
Lymphoma	15 (11.2%)	<15 (16.4%)	*
Soft tissue sarcoma	10 (7.5%)	5 (6.8%)	5 (8.2%)
Other	20 (13.4%)	10 (16.4%)	5 (9.8%)
Missing	5 (4.5%)	*	*

*= Sample size under 5 so data not shown due to privacy concerns.

**Figure 2:**
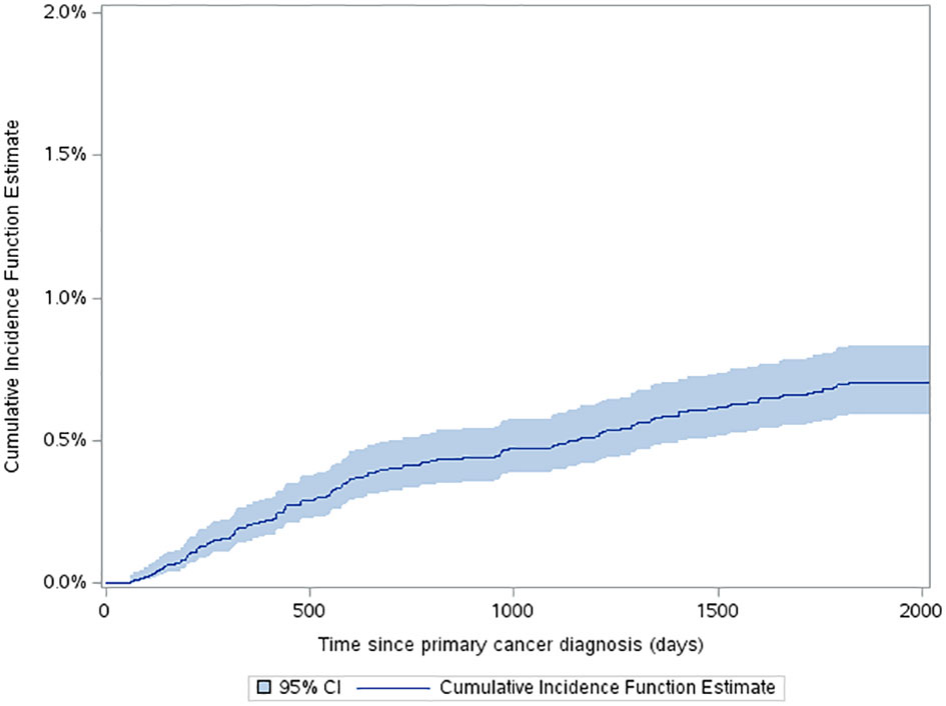
Estimated cumulative incidence function for a SMN over time from first cancer diagnosis.

**Figure 3:**
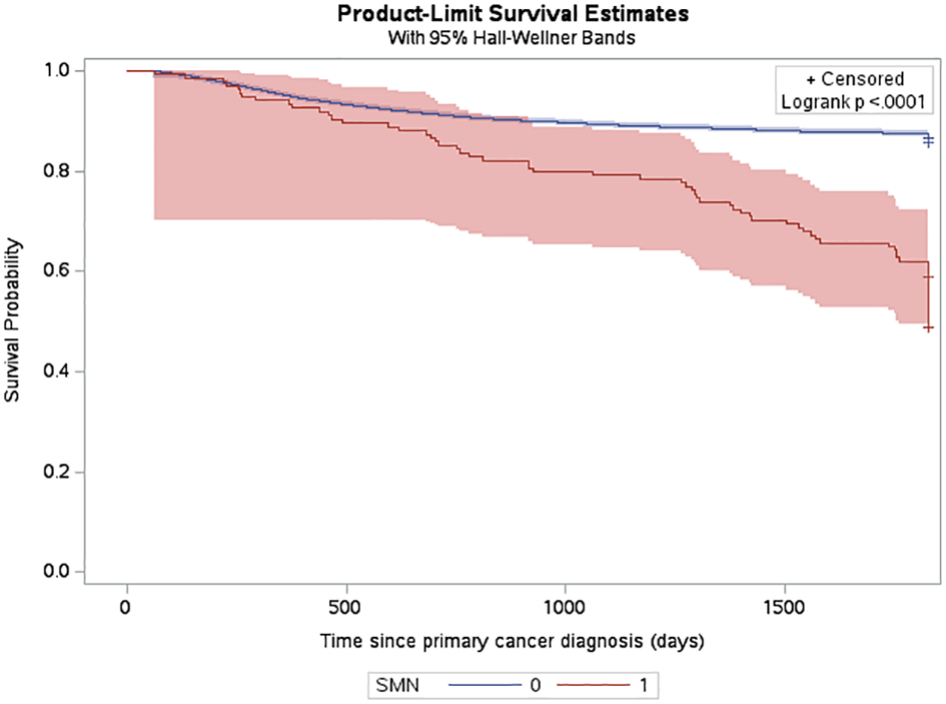
Probability of death among CYP-C patients from 2001-2019 shown by SMN status, censored 5 years after diagnosis or on their death status.

The estimated cumulative incidence function (CIF) for a very-early SMN was 0.42% (95% CI: 0.34% to 0.52%) and this increased to 0.7% (95% CI: 0.59% to 0.83%) by 5 years after diagnosis (**Figure 2**). The three-year probability of survival among patients with a SMN was 79.1% (95% CI: 71.2% to 85.1%) compared to 89.3% (95% CI: 88.8% to 89.7%) in the non-SMN cohort. The five-year probability of survival was 59.0% (95% CI: 50.1% to 66.7%) in the SMN cohort vs. 86.5% (95% CI: 86.0% to 86.9%) among the non-SMN cohort (**Figure 3**).

**Figure 4:**
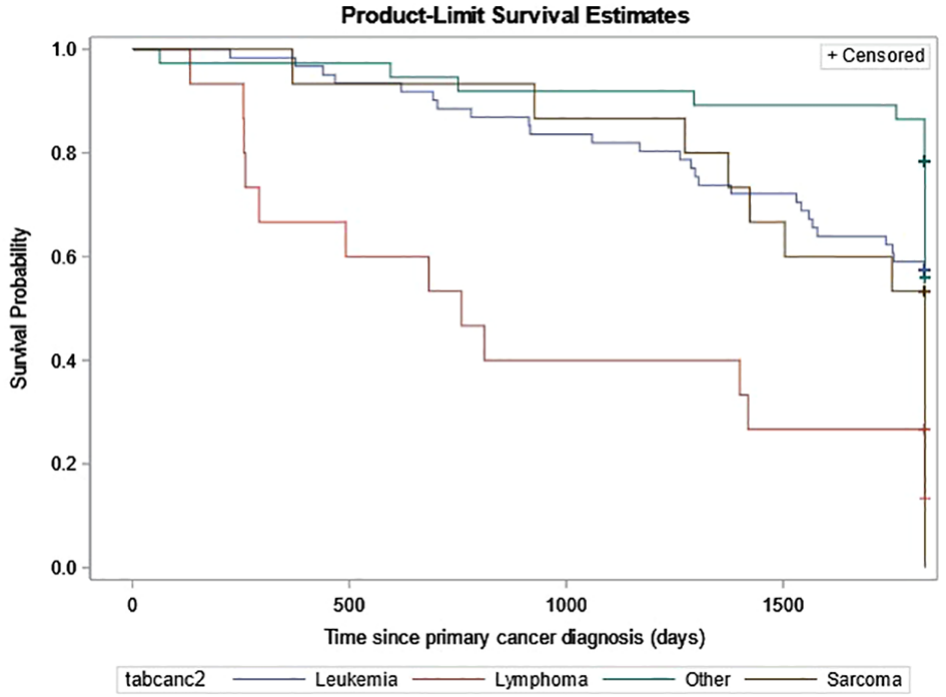
Probability of death among SMNs from 2001-2019 shown by SMN cancer type.

In the full cohort of all first primary cancers, a relapse within 5 years of the first primary cancer was documented in 12.3% of the non-SMN cohort compared to 21.6% of the SMN cohort. Of the 61 patients with leukemia as their SMN diagnosis, 26 patients died. Of those 26 deaths, 20 of them occurred within one year of the SMN diagnosis and the remaining six patients between one to five years. The probability of survival was the lowest for patients diagnosed with lymphoma as their SMN (**Figure 4**).

Appendix 1 shows management of patients with SMN.

### 3.3 Risk factors

**Figure 5:**
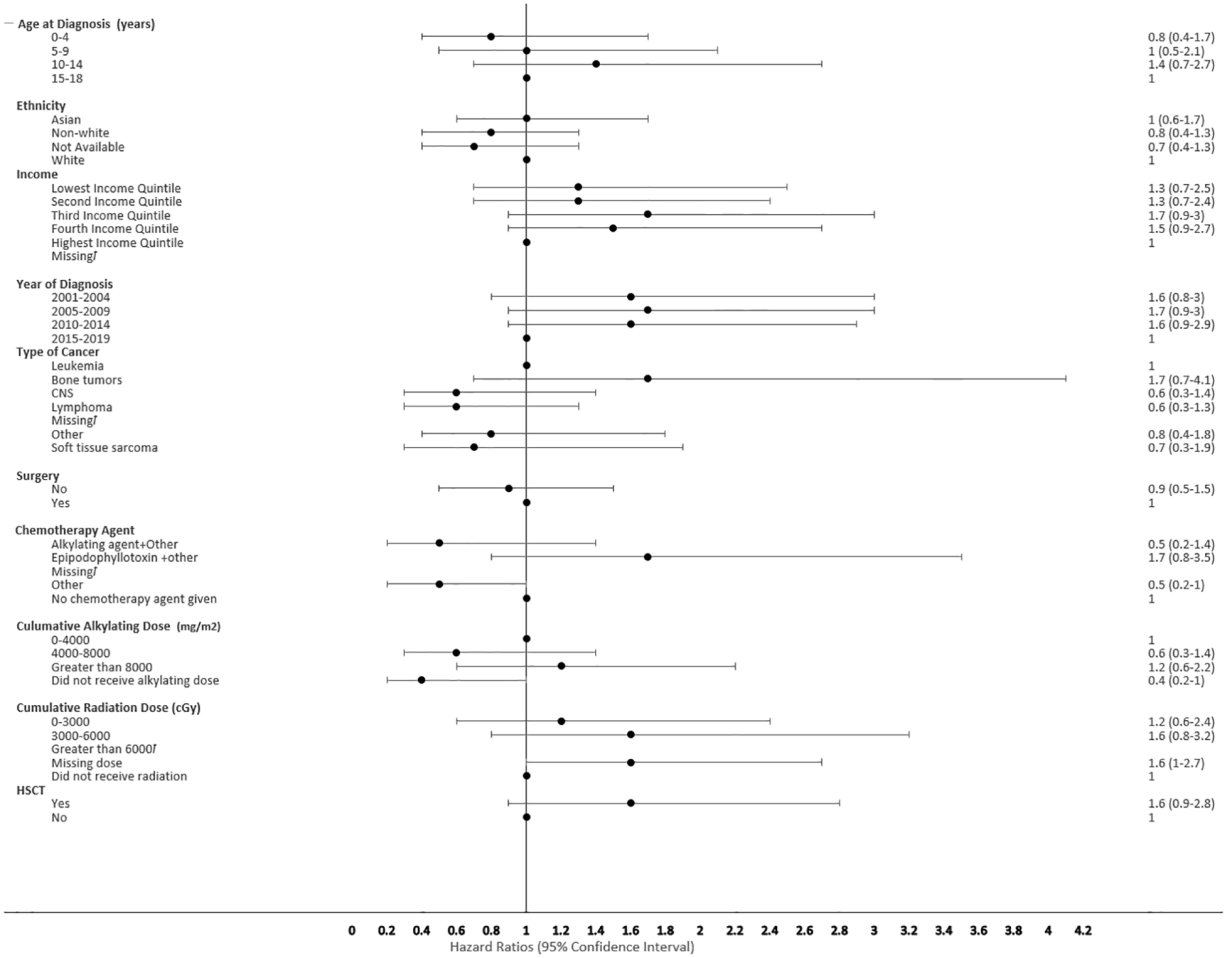
Forest plot depicting the variables and adjusted hazard ratio for SMN. ƚ indicates this hazard ratio has been suppressed because less than 5 patients have the characteristic.

**Figure 6:**
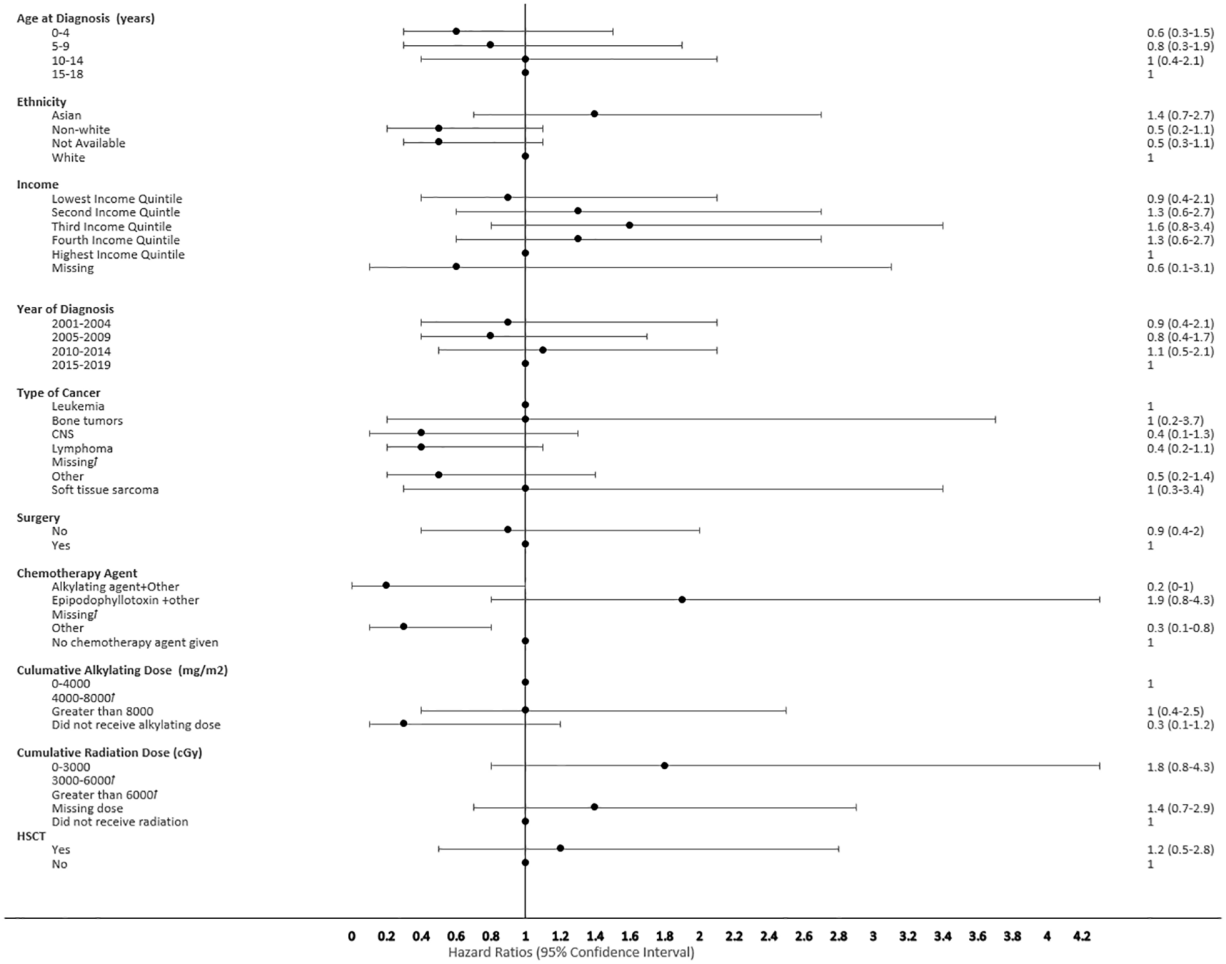
Forest plot depicting the variables and adjusted hazard ratio for SMN developed within 0-2 years. ƚ indicates this hazard ratio has been suppressed because less than 5 patients have the characteristic.

**Figure 7:**
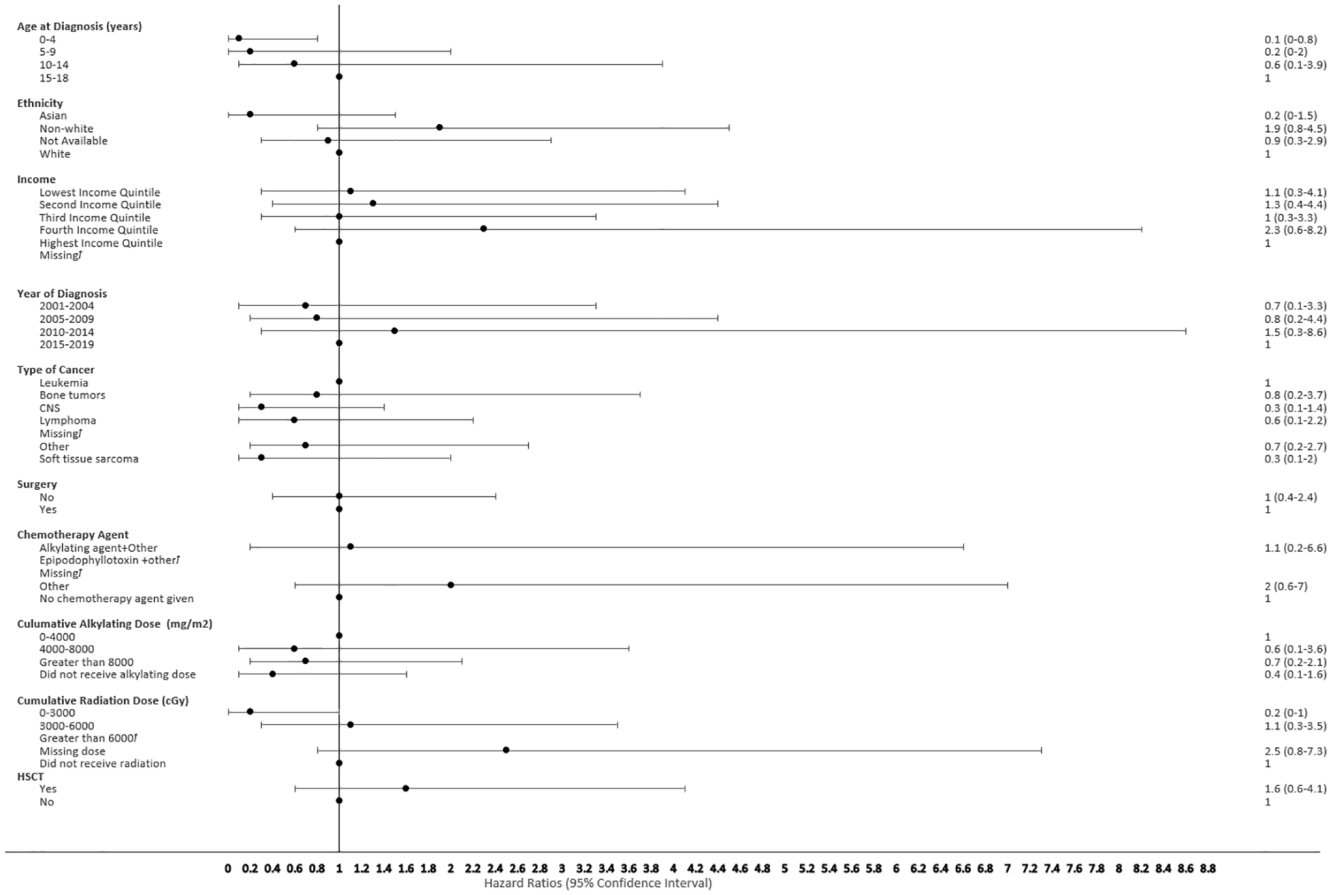
Forest plot depicting the variables and adjusted hazard ratio for SMN developed within 2-5 years. ƚ indicates this hazard ratio has been suppressed because less than 5 patients have the characteristic.

The proportional hazard regression model derived possible risk factors for developing a SMN based on variables identified in **Table 1**, accounting for the competing risk of death. All the hazard ratios were adjusted for HSCT, chemotherapy agent categories in **Table 1**, cancer types, cumulative radiation and alkylating doses, surgery, year of diagnosis, ethnicity, neighborhood income quintile and age at diagnosis. A very few therapeutic exposures reached statistical significance as risk factors. In patients who were 0-4 years old at their initial diagnosis they have a 10% lower hazard [0.1 (95% CI: 0.0-0.8) of developing an early SMN compared to those who were 15-18 years old. In patients who had other chemotherapy treatments in their first diagnosis, they have a 30% lower hazard [0.3 (95% CI: 0.1-0.8) of developing a very-early SMN compared to not having had a chemotherapy treatment. Although not statistically significant, exposure to epipodophyllotoxins [1.7 (95% CI: 0.8 to3.5)], radiation therapy of 3000-6000 cGy [1.6 (95% CI: 0.8 to 3.2)], and/or HSCT [1.6 (95% CI: 0.9 to 2.8)] were clinical risk factors associated with a higher hazard of SMN development compared to their reference groups (**Figure 5**). Alkylating agents and epipodophyllotoxins still constitute major components of many treatment protocols. We saw a trend for very-early onset SMN among patients with exposure to epipodophyllotoxins, this trend was also true in the overall cohort for onset of a SMN (**Figures 5**, **6**). Contrary to this, we saw the use of alkylating agents had a trend of lowering (**Figures 5**, **6**) or no effect (**Figure 7**) on the onset of a SMN. However, the cumulative alkylating dose response relationship was inconsistent with some showing using 4,000-8,000 mg/m^2^ as lowering SMN risk more than using 0-4,000 mg/m^2^ (**Figure 5**). For those who had both radiation and an alkylating agent for their treatment of primary cancer, 1.2% had developed SMN, which is slightly higher than the 1% we see in general population for the development of SMN.

## 4 Discussion

### 4.1 Interpretation of findings

The five-year cumulative probability of SMN in our cohort is comparatively lower than the previous Canadian study published by Pole et al. (22) The cumulative incidence derived from our risk model is likely more conservative due to the following two reasons. Firstly, we used multiple competing risk factors and mutually exclusive outcome variables in our model and secondly, our cohort was more recent; therefore, treated using more risk stratified therapeutic approach and consequently exposed to fewer risk factors like high dose radiation or alkylating agents. A recent pediatric cancer survivor population study by Ju et al. showed a similar estimated five-year cumulative incidence of SMN at 0.7% (2). The incidence of SMN in the 15-18 years old age group is much lower in our study, likely due to their more recent diagnosis (i.e., since January 1, 2015) in an era of risk-stratified therapeutic approaches, less complete follow up, and under-representation of adolescent and young adult (AYA) population within the CYP-C dataset. As previously mentioned, there is a lack of continuity and completeness of data available for this subset of the adolescent population. It is crucial to link pediatric cancer databases with the adult registries to avoid early censure and loss of follow up data among the AYA population.

Risk factors associated with second neoplasms have been well described among the late onset SMN, which is defined as those occurring more than 5 years after the first primary cancer, with exposure to radiation therapy being the major factor. The risk of developing a SMN post-radiation therapy has been described to increase with advancing age of the survivors, but its impact on very-early onset SMN is less well described (35, 36). We also did not see a clear association of early SMNs with radiation, given that a smaller proportion of children received upfront radiation therapy for their first cancer diagnosis. Chemotherapy agents still constitute first line therapy for many pediatric cancers (**Table 1**). HSCT is a well-described risk factor for SMN, especially among recipients of myeloablative conditioning regimens during transplant, and this risk was seen in our cohort (34, 37). Many of the HSCT protocols used total body radiation and/or high dose alkylating agents in myeloablative conditioning regimens, which reiterates the risk of SMN secondary to high dose chemotherapy and radiation therapy (**Figure 5**). Given the multi-modal treatment approach in pediatric cancer, there is a complex and cumulative impact of treatment in the development of a SMN. This potentially could be due to the size of SMN cohort, methods of capturing data and duration of follow up available.

By using a sub-distributional hazard model, the effects of an individual risk co-variate on SMN were analyzed in the context of the competing risk of death. However, the retrospective nature of the study limited us to only propose an association of SMN with individual risk factors such as exposure to epipodophyllotoxins, high dose alkylating agents, radiation, and HSCT toward the development of a SMN. The hazard ratios might be lower compared to the other studies because the model tries to analyze the impact of an individual risk factor amidst competing factors and is not quantifying the synergic interactions possible between multiple risk factors. Though we did not see any collinearity among the factors in our analysis, we are aware that these covariates have a synergistic relationship clinically. Building future models based on mediation analysis can be used to derive an individual’s risk for SMN from independent co-variates, which can potentially be helpful with personalized surveillance recommendations.

### 4.2 Strengths and limitations

This is one of the largest population-surveillance databases based SMN studies in pediatrics that utilized trained scientific personnel input study data, and thereby minimizing the potential risk of selection and recall bias. Extensive data quality improvement steps (e.g., mapping, merging, creating derived variables, etc.) were taken for these data, and the CYP-C diligently attempted to minimize such errors by comprehensive training of data collectors and conducting regular data audits.

Another strength was our modeling approach, which utilized the Fine Grey method where you create a sub-distribution hazard model based on the cumulative incidence function accounting for covariates and the presence of competing risks. Competing risk analysis allows you to better assess censoring in a population where a patient is at risk of more than one mutually exclusive events occurring, such as in a population of pediatric oncology patients.

Despite the strengths of our study, we did not have the ability to look at other important SMN risk factors, such as cumulative dose of epipodophyllotoxins, which have been shown to be associated with therapy-related leukemias. Also, the limited sample size of our study, while large for SMN studies but not large for the general population, created model instability and we were unable to stratify the chemotherapy agents (e.g., anthracyclines) more granularly and they were grouped into another category. Additionally, the CYP-C started to gather data on children aged 15-18 after 2015; however, this data is not comprehensive or complete due to the inconsistency in capturing this patient population by the pediatric cancer centers vs. the adult cancer centers. Therefore, data for this age group should be interpreted with caution. The high frequency of SMN among bone tumors could be associated with cyclophosphamide (38). Additionally, although 17.1% of pediatric first primary cancer patients and 26.9% of SMN patients had an underlying genetic cancer predisposition, this may be an underestimation as not all patients had genetic testing. Another limitation of the study is the low yield of CYP-C data in differentiating histologically or morphologically distinct SMN from the primary cancer. Future studies with sufficient treatment data should investigate the association of anthracyclines in SMN risk more closely. Finally, although many efforts were made to increase the accuracy of findings, the study did not consider the interactions between the risk factors. Therefore, future research should include those interactions to enhance the understanding of the overall risk. Currently, there are no large-scale Canadian studies on the early SMN among survivors of childhood cancer. Our study adds valuable data and information from the Canadian perspective to the existing literature on this topic. Furthermore, this data will assist both short term and long term follow up clinics in Canada to maintain heightened surveillance for SMNs in high-risk categories of patients.

### 4.3 Clinical takeaways

Though the cumulative incidence of SMN has decreased, the five-year survival outcome in our early onset SMN cohort has not significantly improved compared to previously published data (39). Acute leukemia constituted more than 50% of our study cohort, with acute myeloid leukemia being the most common type of SMN in the first 2 years. Although previous literature indicated thyroid cancer as one of the most common SMNs (5), we still consider leukemia as the most common SMN as this was considered with or without receiving radiation therapy for the first cancer. The historical outcomes for early onset SMN with hematological malignancies have been poor (40, 41). Given the inconsistent nature of pediatric cancer centers’ long-term monitoring and surveillance of mortality rates and the quality of life of pediatric cancer survivors across the nation, we, subsequently, also have insufficient data on the adolescent population. Our data suggests the need to incorporate awareness and surveillance for SMN within 5 years of diagnosis in follow-up clinics across Canada. The linking of pediatric cancer databases with the adult registries will allow a better systematic approach to surveillance and follow-up among the adolescent and young adults population.

## Data availability statement

The datasets presented in this article are not readily available because individual participant data cannot be shared due to Cancer in Young People in Canada policies. Requests to access the datasets should be directed to the C17 Council website, available at https://www.c17.ca/index.php?cID=70 (login required).

## Ethics statement

The studies involving humans were approved by IWK Health Research Ethics Board. The studies were conducted in accordance with the local legislation and institutional requirements. Written informed consent for participation was not required from the participants or the participants’ legal guardians/next of kin in accordance with the national legislation and institutional requirements.

## Author contributions

CR: Data curation, Formal analysis, Funding acquisition, Investigation, Methodology, Writing – original draft, Writing – review & editing. KL: Writing – original draft, Writing – review & editing. DS: Formal analysis, Writing – original draft, Writing – review & editing. NS: Data curation, Writing – review & editing. JK: Data curation, Writing – review & editing. LX: Writing – review & editing. ML: Writing – review & editing. DZ: Writing – review & editing. JP: Writing – review & editing. M-CP-M: Writing – review & editing. RB: Writing – review & editing. SI: Writing – review & editing. T-HT: Writing – review & editing. SO: Writing – review & editing. SR: Writing – review & editing. TM: Writing – review & editing. LS: Writing – review & editing. KK: Conceptualization, Data curation, Investigation, Methodology, Project administration, Resources, Supervision, Validation, Writing – original draft, Writing – review & editing.

## Appendix 1 Management of SMN cancer for the whole SMN cohort.

**Table:**

	Chemotherapy	Radiation	Surgery	HSCT / Bone Marrow Transplant	Clinical Trial
ALL (n=7)	6	<5	<5	0	<5
AML (n=31)	26	14	17	<5	7
Other Leukemia (n=23)	20	12	8	11	9
Bone (n=5)	5	<5	<5	<5	<5
CNS (n=19)	9	7	9	<5	<5
Lymphoma (n=15)	13	7	10	<5	5
Other Cancers (n=18)	9	5	10	0	<5
Soft Tissue (n=10)	10	<5	8	<5	<5
TOTAL	104	54	69	30	35
