# Corrigendum: Second malignant neoplasms within 5 years from first primary diagnosis in pediatric oncology patients in Canada: a population-based retrospective cohort study

**DOI:** 10.3389/fonc.2024.1418512

**Published:** 2024-05-10

**Authors:** Christina Ricci, Divya Subburaj, Kate Lim, Neetu Shukla, Jaskiran Kaur, Lin Xie, Meghan Laverty, Dianne Zakaria, Jason Pole, Marie-Claude Pelland-Marcotte, Randy Barber, Sara J. Israels, Thai-Hoa Tran, Sapna Oberoi, Samuele Renzi, Tamara MacDonald, Lillian Sung, Ketan Kulkarni

**Affiliations:** ^1^ Lifespan Chronic Disease and Conditions Division, Public Health Agency of Canada, Ottawa, ON, Canada; ^2^ Department of Pediatrics, Division of Hematology-Oncology, Izzak Walton Killam (IWK) Health Centre, Halifax, NS, Canada; ^3^ Faculty of Medicine, Dalhousie University, Halifax, NS, Canada; ^4^ Surveillance Systems and Data Management Division, Public Health Agency of Canada, Ottawa, ON, Canada; ^5^ Dalla Lana School of Public Health, University of Toronto, Toronto, ON, Canada; ^6^ Centre for Health Sciences Research, University of Queensland, Brisbane, QLD, Australia; ^7^ Division of Pediatric Hematology-Oncology, CHU de Québec-Centre Mère-Enfant Soleil, Quebec City, QC, Canada; ^8^ Research Centre of the CHU de Québec, Axe Reproduction, Santé de la Mère et de l’Enfant, Quebec City, QC, Canada; ^9^ C17 Research Network, C17 Council, Edmonton, AB, Canada; ^10^ Department of Pediatrics and Child Health, University of Manitoba, Winnipeg, MB, Canada; ^11^ Department of Pediatrics, Division of Pediatric Hematology-Oncology, Charles-Bruneau Cancer Center, Centre Hospitalier Universitaire (CHU) Sainte-Justine, Montréal, QC, Canada; ^12^ Immune Diseases and Cancers Axis, CHU Sainte-Justine Research Center, Montréal, QC, Canada; ^13^ Department of Pediatric Hematology-Oncology, CancerCare Manitoba, Winnipeg, MB, Canada; ^14^ Division of Hematology Oncology, The Hospital for Sick Children, Toronto, ON, Canada; ^15^ Department of Anesthesia and Intensive Care, IRCCS San Raffaele Scientific Institute, Milan, Italy; ^16^ Department of Pharmacy, Izzak Walton Killam (IWK) Health, Halifax, NS, Canada; ^17^ Faculty of Health Professions, Dalhousie University, Halifax, NS, Canada; ^18^ Child Health Evaluative Sciences, Research Institute, The Hospital for Sick Children, Toronto, ON, Canada

**Keywords:** oncology, pediatrics, risk factors, early second malignant neoplasm (early SMN), Canada, surveillance

In the published article, there was an error in the legend for **Figure 5** to **7** as published. Currently it stated “This hazard ratio has been suppressed because less than 5 patients have the characteristic.” The corrected legend appears below.

“ƚ indicates this hazard ratio has been suppressed because less than 5 patients have the characteristic.”

**Figure 5:**
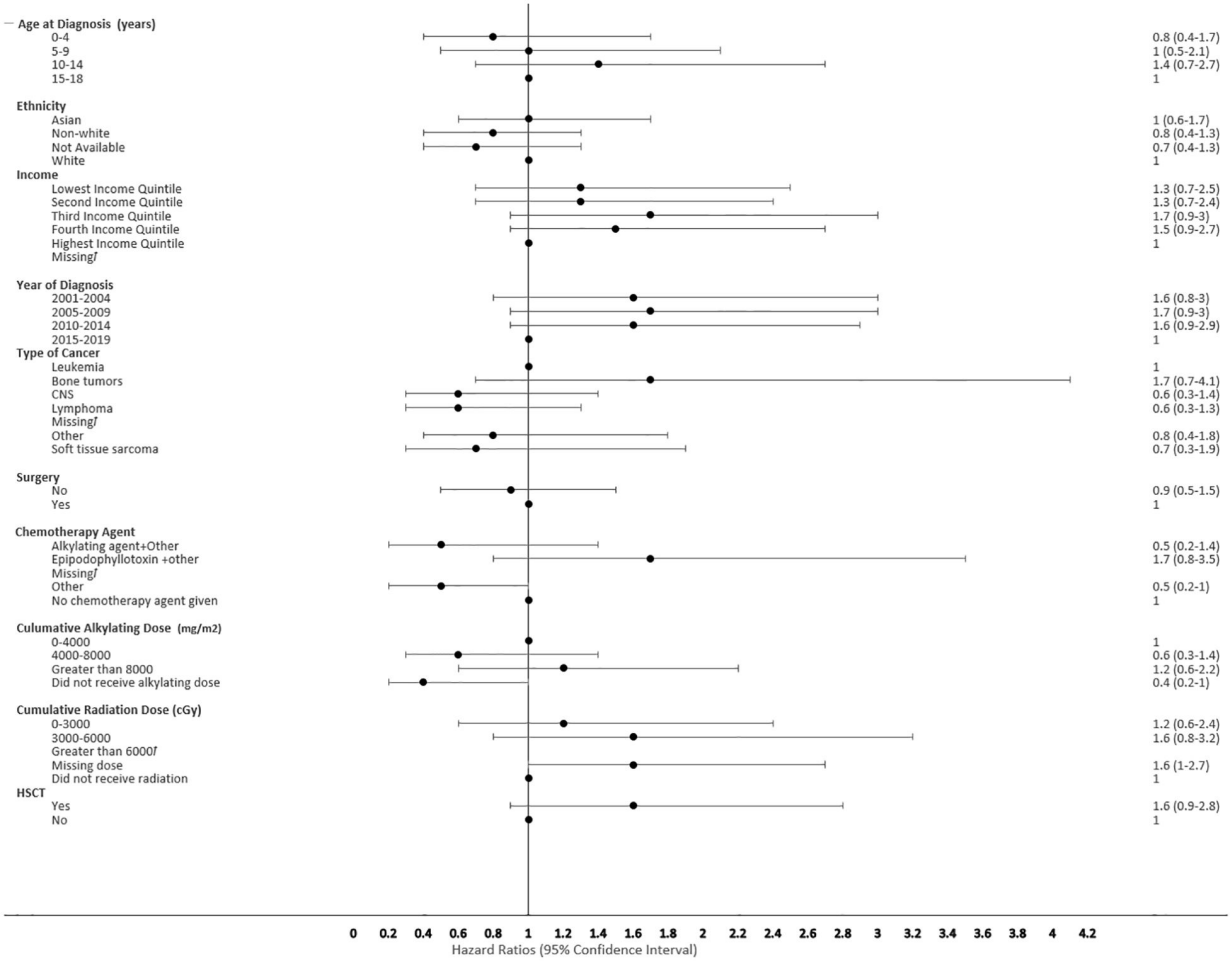
Forest plot depicting the variables and adjusted hazard ratio for SMN. ƚ indicates this hazard ratio has been suppressed because less than 5 patients have the characteristic.

**Figure 6:**
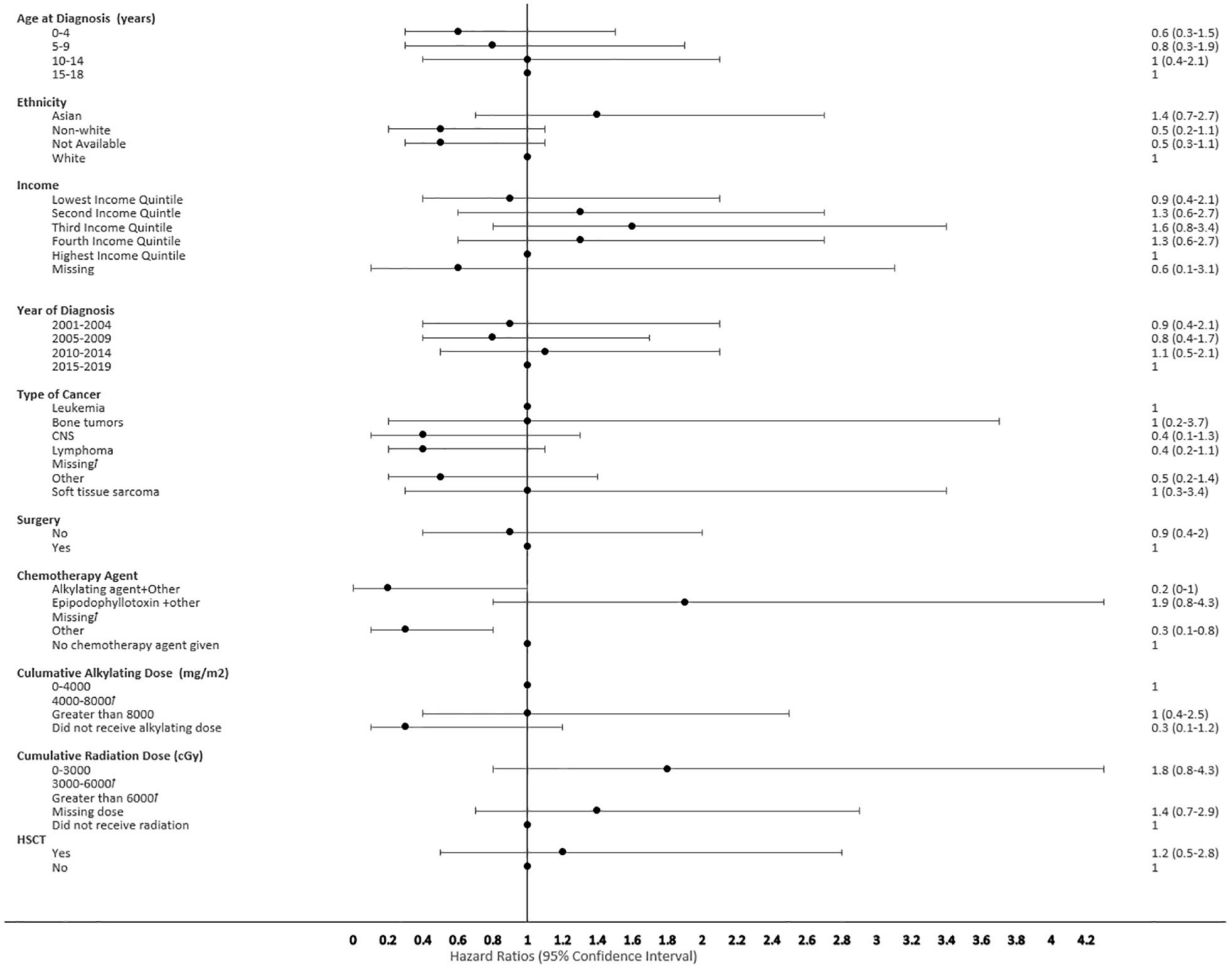
Forest plot depicting the variables and adjusted hazard ratio for SMN developed within 0-2 years. ƚ indicates this hazard ratio has been suppressed because less than 5 patients have the characteristic.

**Figure 7:**
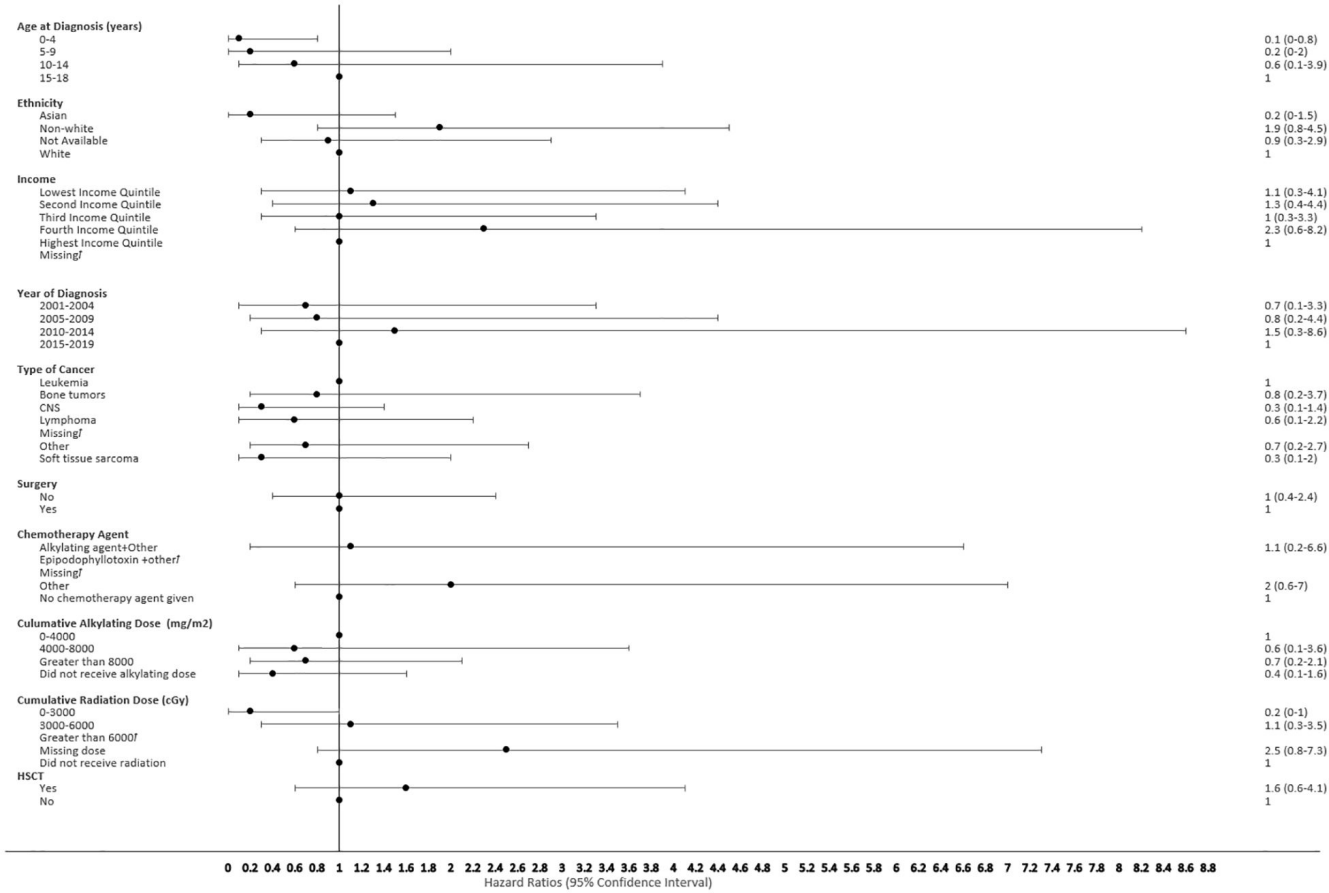
Forest plot depicting the variables and adjusted hazard ratio for SMN developed within 2-5 years. ƚ indicates this hazard ratio has been suppressed because less than 5 patients have the characteristic.

The authors apologize for this error and state that this does not change the scientific conclusions of the article in any way. The original article has been updated.

